# Preclinical Safety Profile of Deg-AZM, a Clinical-Stage New Transgelin Agonist: hERG Inhibition Study In Vitro, Cardiovascular–Respiratory Pharmacology, and Single/Repeated-Dose Toxicity in Beagle Dogs

**DOI:** 10.3390/biomedicines13092180

**Published:** 2025-09-06

**Authors:** Xiaoting Gu, Xiaohe Li, Hailong Li, Nannan Liu, Ying Xu, Keran Li, Jia Zhang, Xiaoting Wang, Xiaoting Zhang, Yanjie Ding, Honggang Zhou, Xiaoyu Ai, Cheng Yang

**Affiliations:** State Key Laboratory of Medicinal Chemical Biology, College of Pharmacy and Tianjin Key Laboratory of Molecular Drug Research, Nankai University, Tianjin 300350, China; guxiaoting@nankai.edu.cn (X.G.); lixiaohe908@163.com (X.L.); hailongli@mail.nankai.edu.cn (H.L.); 18032422509@163.com (N.L.); xyying1688@163.com (Y.X.); lkr13111337893@163.com (K.L.); zhangjia2001@126.com (J.Z.); 15606406360@163.com (X.W.); zhangxiaoting066@126.com (X.Z.); 18632287161@126.com (Y.D.); honggang.zhou@nankai.edu.cn (H.Z.)

**Keywords:** Deg-AZM, preclinical toxicology, hERG inhibition, cardiovascular–respiratory pharmacology, acute toxicity, repeated toxicity

## Abstract

**Background:** Slow transit constipation (STC) represents a refractory gastrointestinal disorder with limited therapeutic options. Deglycosylated azithromycin (Deg-AZM) is a small molecule Transgelin agonist effective against STC, which has been approved for 2024 clinical trials. **Objectives:** This study comprehensively evaluated the cardiac safety (hERG inhibition), acute cardiovascular–respiratory effects, and single/repeated-dose toxicity of Deg-AZM in Beagle dogs to de-risk clinical translation. **Methods:** Using automated patch-clamp (hERG-HEK293 cells; 0.1–1000 μM), telemetric monitoring in Beagles (3/8/24 mg/kg; Latin square design), and GLP-compliant toxicity studies (single-dose: 150–300 mg/kg; 28-day: 5–50 mg/kg/day), we assessed functional, biochemical, histopathological, and toxicokinetic parameters. **Results:** Deg-AZM showed negligible hERG inhibition (maximum 21.3% at 1000 μM). Transient PR prolongation (24 mg/kg; resolved by 4 h) and respiratory rate reduction (8–24 mg/kg; resolved by 2 h) occurred at supratherapeutic doses. Single-dose toxicity revealed one mortality at 300 mg/kg (acute cardiac ischemia), while 28-day studies identified fully reversible myocardial vacuolation at 50 mg/kg. Toxicokinetics demonstrated dose-proportional exposure (AUC and C_max_) and low accumulation (accumulation factors ≤ 1.5). No hematological, coagulation, or hepatic toxicity was observed. **Conclusions:** With absent hERG liability and manageable transient physiological effects, Deg-AZM exhibited a favorable preclinical safety profile supporting its clinical development for STC.

## 1. Introduction

Slow transit constipation (STC), characterized by significantly delayed colonic propulsion due to impaired motility, represents a prevalent and refractory subtype of functional constipation. It manifests clinically as infrequent bowel movements, straining, hard stools, abdominal bloating, and significantly diminished quality of life [1,2]. The global incidence of constipation, including STC, continues to rise, driven by factors such as dietary changes, sedentary lifestyles, and aging populations [3,4]. Despite its significant burden, STC pathogenesis involves complex and multifactorial mechanisms, leading to a lack of universally effective treatments [5,6]. Current first-line management relies on conservative measures (laxatives, dietary fiber, lifestyle modification) [7,8], while surgical interventions like colectomy are reserved for severe, unresponsive cases but carry substantial risks of complications such as persistent symptoms, diarrhea, and bowel obstruction [9,10]. This unmet clinical need underscores the urgency for developing novel, efficacious, and safer pharmacological agents specifically targeting the underlying dysmotility in STC.

Azithromycin (AZM), a macrolide antibiotic, has been recognized for its prokinetic effects in gastrointestinal motility disorders [11,12,13]. However, its utility as a chronic therapy for constipation is precluded by its antibiotic activity and associated side effects, particularly gastrointestinal irritation with significant inter-individual variability [14,15,16]. Interestingly, deglycosylated azithromycin (Deg-AZM), a unique metabolite of AZM devoid of its parent compound’s antibacterial properties, has emerged as a promising candidate. Our previous research identified Deg-AZM as a potent positive intestinal agonist, effectively stimulating intestinal contractility [17]. Mechanistically, Deg-AZM functions as a novel small molecule agonist of Transgelin (also known as SM22α), a 22-kDa actin-binding protein within the Calponin family abundantly expressed in smooth muscle, including the gut [18,19,20]. Deg-AZM promotes intestinal peristalsis by upregulating Transgelin expression in intestinal smooth muscle cells, facilitating the polymerization of globular actin (G-actin) into filamentous actin (F-actin), and enhancing the formation of contractile stress fiber bundles [18]. As the first-in-class Transgelin agonist developed specifically for STC, Deg-AZM holds significant therapeutic potential and has received implied approval for clinical trials in China (acceptance number CXHL2400005).

Prior to clinical progression, comprehensive preclinical safety evaluation is paramount. Potential cardiac toxicity, particularly, inhibition of the human ether-à-go-go-related gene (hERG) potassium channel (associated with life-threatening arrhythmias like Torsades de Pointes), is a critical concern for any new chemical entity [21,22]. Furthermore, detailed assessment of effects on vital cardiovascular and respiratory functions in conscious large animals, alongside thorough characterization of toxicity profiles (including single-dose lethality and repeated-dose toxicity in a relevant species), is essential to define safety margins and guide clinical dosing [23,24]. Beagle dogs represent a standard non-rodent species for such safety pharmacology and toxicology studies due to their physiological similarities to humans in cardiovascular and gastrointestinal systems.

The aim of this study was to investigate the comprehensive preclinical safety assessment of Deg-AZM. The potential to inhibit the hERG potassium channel was evaluated in vitro, acute effects on cardiovascular and respiratory parameters were investigated in conscious telemetered Beagle dogs, and single-dose/28-day repeated-dose toxicity profiles were determined in Beagle dogs. These investigations are crucial for establishing the initial safety profile of this novel Transgelin agonist and informing its risk–benefit assessment in future clinical trials for STC.

## 2. Materials and Methods

### 2.1. Chemicals, Reagents, and Materials

Deg-AZM was obtained from Nankai University (Tianjin, China). Cisapride was provided by Sigma-Aldrich Pty Ltd. (Saint Louis, MO, USA). All of the reference substances were 98% purity. Carboxymethylcellulose sodium (CMC-Na) was obtained from Sinopharm Chemical Reagent Co., Ltd. (Shanghai, China). Deionized water used throughout the study was provided by Wahaha Corporation (Hangzhou, China).

### 2.2. Animals

The Beagle dog is widely used in pharmacological and toxicological experiments due to its well-established background data, consistent historical use both domestically and internationally, and readily available domestic supply. Therefore, this breed was selected for the safety evaluation of Deg-AZM. Beagle dogs, aged 6 to 8 months with body weights ranging from 8.12–9.05 kg for males and 7.07–8.40 kg for females, were obtained from Weifang Shengnuo Laboratory Animal Breeding Co., Ltd., Weifang, China. The dogs used in the cardiovascular and respiratory safety pharmacology studies, toxicokinetic studies were fasted overnight prior to the study initiation. All animals were maintained on a standard diet of canine maintenance compound feed. Throughout the study, the housing environment was maintained at 15–29 °C with 40–62% relative humidity, complying with standard animal welfare conditions. Beagle dogs that passed quarantine were used for subsequent research. All animal studies were designed and reported in accordance with the ARRIVE guidelines. All procedures in this study were performed in animal experimental facilities certified by the International Laboratory Animal Evaluation and Certification Management Committee, and approved by the Institutional Animal Care and Use Committee at Shandong Xibo Drug Safety Evaluation Research Center (XB-IACUC-2022-1600). Sample sizes were determined based on OECD Guideline 409.

### 2.3. hERG Channel Inhibition Assay

The stably transfected hERG-expressing cell line (hERG-HEK293) was kindly provided by the New Drug Safety Evaluation Research Center of Hebei Medical University. The hERG-HEK293 cell line stably expressing hERG potassium channels was cultured in DMEM/F12 medium containing 10% FBS and 0.2 mg/mL G418. Whole-cell patch-clamp recordings were performed using an automated Patchliner^®^ system (Nanion Technologies, Munich, Germany) with EPC 10 Quadro amplifier. Cells were perfused with extracellular solution containing either Deg-AZM (0.1–1000 μM) or cisapride (positive control, 0.01–3 μM) in ascending concentrations. The voltage protocol consisted of depolarization to +30 mV for 2.5 s from a –80 mV holding potential, followed by repolarization to –50 mV for 4 s to elicit tail currents. Current amplitudes were normalized to vehicle control (100%), with concentration–response curves and IC_50_ values generated using Igor Pro 9.0 (Nanion) and GraphPad Prism 10.1.

### 2.4. Cardiovascular and Respiratory Safety Pharmacology Study in Beagle Dogs

Eight healthy Beagle dogs (4 males/4 females) were assigned using a Latin square design to receive Deg-AZM via oral gavage across four treatment cycles, with 6-day washout intervals between cycles. Following overnight fasting, dogs were administered Deg-AZM suspensions at 3, 8, or 24 mg/kg or vehicle control (0.5% CMC-Na). Non-invasive physiological parameters were continuously monitored using EMKA telemetry jackets (emkaPack4G system with iox v2.9.5 software, EMKA Technologies, Paris, France) for blood pressure (systolic blood pressure, diastolic blood pressure, and mean arterial pressure), lead II ECG (HR, P/R/T waves, PR/ST/QT/QTc intervals, QRS complex), and respiratory function (respiratory rate, tidal volume, maximal lung capacity). Data were analyzed at pre-dose and 0.5, 1, 2, 4, 8, and 24 h post-dose using ecgAUTO 6.0 software. Clinical signs and mortality were recorded daily during acclimation and trial periods via cage-side observation. Body weights were measured pre-fasting on selection days and pre-dose on treatment days using a TCS-150B electronic scale (Zhongshan Camry Electronic Scale Co., Ltd., Zhongshan, China).

### 2.5. Single-Dose Toxicity Study of Oral Deg-AZM Administration in Beagle Dogs

Eight healthy Beagle dogs (4 males/4 females) were randomly divided by sex and body weight into vehicle control group (0.5% CMC-Na) and Deg-AZM group. This experiment employed a cumulative dosing regimen with sequential dose escalation (150, 225, and 300 mg/kg) administered at 24-h intervals until either >50% mortality was observed or the maximum dose level was reached. Surviving animals underwent a 14-day recovery period following the final administration. The minimum lethal dose (MLD) and median lethal dose (LD_50_) were determined as follows: (1) when no mortality occurred, both MLD and LD_50_ were considered greater than the highest tested or limit dose; (2) when complete mortality was observed at a given dose level, MLD and LD_50_ were estimated between this dose and the preceding lower dose; (3) when partial mortality occurred at one dose with additional deaths at the subsequent higher dose, MLD was established between the first lethal dose and its immediate predecessor, while LD_50_ was calculated between the initial lethal dose and the dose producing 100% mortality.

#### 2.5.1. Clinical Observations

Animals were observed continuously on dosing days, and once each in the morning and afternoon on non-dosing days, with all surviving animals included in the assessment. On dosing days, observations were conducted at fixed intervals before and for 6 h after dosing, with detailed recordings at 5, 10, 20, 30, 40, and 50 min, as well as 1, 1.5, 2, 3, 4, 5, and 6 h post-dosing. Any symptoms at other time points were noted in the records. On non-dosing days, detailed observations were performed twice daily to assess general reactions, activity levels, and overall condition, including appearance, behavior, food intake, response to stimuli, secretions, excretions, and mortality. Toxic manifestations, onset and resolution of adverse effects, and time of death were recorded. Body weight was measured in all surviving animals upon arrival, before grouping/prior to each dosing, and on recovery days 3, 7, 10, and 14. Food intake was monitored daily in all surviving animals by providing each dog with 125 ± 5 g of pelleted feed in the morning and afternoon, with leftovers checked the following morning. On dosing days, feeding occurred no sooner than 1 h post-administration.

#### 2.5.2. Electrocardiographic Examination

Electrocardiogram (ECG) assessments were performed at the following time points: once before dosing (baseline), on the day following each administration, on day 14 of the recovery period, and immediately for any moribund animals. All surviving animals were included in the analysis. Standard lead II ECGs were recorded using an ECG Processor EP-2B electrocardiograph (Nihon Kohden, Tokyo, Japan), with measurements including heart rate (HR), P wave, R wave, T wave, PR interval, and QT interval, followed by calculation of the corrected QT interval (QTc).

#### 2.5.3. Hematological Examination

Hematological parameters were assessed at baseline, on the day following each administration, on day 14 of the recovery period, and immediately for any moribund animals, with all surviving animals included in the analysis. Evaluated parameters included red blood cell count (RBC), hematocrit (HCT), hemoglobin concentration (HGB), mean corpuscular volume (MCV), mean corpuscular hemoglobin (MCH), mean corpuscular hemoglobin concentration (MCHC), red cell distribution width (RDW), white blood cell count (WBC), platelet count (PLT), platelet distribution width (PDW), leukocyte differential count and percentages (LYM%, NEU%, BASO%, EOS%, MONO%, and absolute counts LYM, NEU, BASO, EOS, MONO), and reticulocyte percentage (RETIC%). Approximately 0.5 mL of venous blood of Beagle dogs was collected from limb veins into EDTA-anticoagulant tubes and analyzed by the clinical laboratory using an ADVIA 2120 hematology analyzer (Siemens Healthineers, Shanghai, China).

#### 2.5.4. Serum Biochemical Analysis

Serum biochemical parameters were evaluated at baseline, on the day following each administration, on day 14 of the recovery period, and immediately for any moribund animals, with all surviving animals included in the analysis. Assessed parameters included aspartate aminotransferase (AST), alanine aminotransferase (ALT), alkaline phosphatase (ALP), total bilirubin (TBIL), total protein (TP), albumin (ALB), globulin (GLO), albumin-to-globulin ratio (A/G), blood urea nitrogen (BUN), creatinine (CREA), total cholesterol (CHOL), triglycerides (TGL), glucose (GLU), creatine kinase (CK), gamma-glutamyl transferase (GGT), and electrolytes (potassium [K], sodium [Na], chloride [Cl], calcium [Ca], phosphorus [PHOS]). Approximately 2 mL of venous blood was collected from limb veins into anticoagulant-free plastic tubes, allowed to clot at room temperature, and centrifuged at 3000 rpm for 10 min to obtain serum for clinical laboratory analysis using a Dimension RxL Max fully automated biochemistry analyzer (Siemens Healthineers, Shanghai, China).

#### 2.5.5. Toxicokinetic Analysis in Single-Dose Toxicity Study

Following single oral administrations of Deg-AZM at 150, 225, and 300 mg/kg to dogs in single-dose toxicity study, toxicokinetic blood sampling was performed for each administration, with approximately 0.5 mL of venous blood collected from canine limbs at each time point: pre-dose and 5, 15, 30 min, 1, 2, 4, 6, 8, 10, and 24 h post-dose. Blood samples were placed in heparinized tubes, centrifuged at 12,000 rpm for 3 min to separate plasma, which was stored at –70 °C until analysis.

Non-compartmental pharmacokinetic analysis was performed on individual concentration-time profiles using DAS 3.3 software (Chinese Pharmacological Association, China). Key parameters assessed included T_max_ (time to maximum concentration), C_max_ (peak concentration), AUC (area under the concentration-time curve), and T_1/2_ (terminal elimination half-life).

### 2.6. 28-Day Repeated Dose Oral Toxicity Study of Deg-AZM in Beagle Dogs

Forty healthy Beagle dogs (20 males/20 females) were randomly divided by sex and body weight into CTL group and different dose groups of Deg-AZM (5, 15, and 50 mg/kg groups). The animals were administered test articles via oral gavage, with the CTL group receiving 0.5% CMC-Na solution and the Deg-AZM groups receiving Deg-AZM at doses of 5, 15, and 50 mg/kg/day. The dosing regimen continued for 28 consecutive days with single daily morning administrations at a volume of 5 mL/kg, followed by a 28-day recovery period.

#### 2.6.1. Toxicological Observations

During the quarantine and acclimation period, Beagle dogs were observed once daily for clinical signs. Following initiation of dosing, animals were monitored three times daily on dosing days (pre-dose in the morning, post-dose between 20–60 min, and in the afternoon) and twice daily on non-dosing days (morning and afternoon). Comprehensive observations included general responsiveness, activity levels, and any signs of toxicity, with detailed documentation of appearance, physical signs, behavioral activities, food consumption, response to stimuli, glandular secretions, respiratory patterns, excretions, fecal characteristics, and mortality. Body weights were recorded weekly during acclimation and twice weekly during the dosing period for all surviving animals. Food consumption was assessed daily by providing each dog with 125 ± 5 g of pelleted diet both in the morning and afternoon, with remaining feed quantified the following morning. Ophthalmic examinations were performed using a YZ25B binocular indirect ophthalmoscope (Suzhou 66 Vision Tech Co., Ltd., Suzhou, China) at pre-dose, end of treatment, and end of recovery, while body temperature measurements were obtained using an Omron MC-510 infrared ear thermometer (Omron Healthcare Co., Ltd., Kyoto, Japan) at baseline, on days 1, 8, 15, and 22 of treatment, immediately after final dose administration (1–2 h post-dose), and at end of recovery.

#### 2.6.2. Electrocardiographic Examination

ECG assessments were performed at the following time points: morning during pre-dose, approximately 1–2 h post-dose on day 15, approximately 1–2 h after the 27th dose administration, and on the morning of recovery period completion. The methodology adhered to the protocol detailed in Section 2.5.2.

#### 2.6.3. Hematological Examination and Coagulation Tests

Hematological parameters were assessed at pre-dose, mid-treatment (Day 15), end of treatment, and end of recovery period. Evaluated parameters and methodology followed the protocol detailed in Section 2.5.3.

Coagulation tests were performed at pre-dose, mid-treatment (Day 15), end of treatment, and end of recovery period. Approximately 1 mL of blood was collected from the limb veins of Beagle dogs and transferred to sodium citrate anticoagulant tubes. Plasma was obtained by centrifugation at 3000 rpm for 10 min, and coagulation parameters including prothrombin time (PT), activated partial thromboplastin time (APTT), thrombin time (TT), and fibrinogen (FIB) were analyzed using an ACL TOP 300 CTS automated coagulation analyzer (Werfen, Bedford, MA, USA).

#### 2.6.4. Serum Biochemical Analysis

Serum biochemical parameters were evaluated at pre-dose, mid-treatment (Day 15), end of treatment, and end of recovery period. Evaluated parameters and methodology followed the protocol detailed in Section 2.5.4.

#### 2.6.5. Toxicokinetic Analysis in 28-Day Repeated Dose Oral Toxicity Study

Toxicokinetic blood sampling was performed during both the initial and final dose administrations in animals that received Deg-AZM via oral gavage at doses of 5, 15, and 50 mg/kg/day as part of the 28-day repeated-dose regimen. The methodology for sample collection, processing, and analysis was identical to that described in detail in “Section 2.5.5 Toxicokinetic Analysis in Single-Dose Toxicity Study”.

#### 2.6.6. Pathological Examination

Pathological examinations were conducted on six animals per group (gender-balanced) at treatment termination and four animals per group (gender-balanced) after the recovery period. Following anesthesia with 7.5 mg/kg tiletamine-zolazepam hydrochloride, animals were euthanized by exsanguination and subjected to gross examination. Absolute organ weights (brain, lungs, thymus, heart, liver [including gallbladder], spleen, kidneys, adrenals, testes, epididymides, ovaries, uterus, and thyroid [including parathyroid]) were measured for organ-to-body weight ratio calculation (g/kg). All weighed organs along with spinal cord (cervical, thoracic, lumbar segments), pituitary, trachea, esophagus, submandibular glands, stomach, duodenum, jejunum, ileum, cecum, colon, rectum, pancreas, aorta, eyes, oviducts, prostate, abdominal skin, mammary glands (females), vagina, sciatic nerves, bladder, optic nerves, sternum (with marrow), femur (with marrow), skeletal muscle, submandibular and mesenteric lymph nodes, tongue, and any macroscopically altered tissues were collected. Eyes and male reproductive organs were fixed in Davidson’s solution for 24 h before transfer to 10% neutral buffered formalin (NBF), while other tissues were directly fixed in 10% NBF. All specimens were paraffin-embedded, sectioned, and stained with hematoxylin and eosin (H&E) for microscopic evaluation.

### 2.7. Data Statistics Method

Animal handling and sample collection were conducted by a single researcher, preventing blinding during these procedures. However, data analysis was performed blindly whenever feasible. Experimental data were plotted using GraphPad Prism 10.1 (GraphPad, San Diego, CA, USA) and expressed as mean ± standard deviation. For body weight, ECG, hematology, coagulation, and serum biochemistry parameters, statistical comparisons between test and vehicle control groups were performed using t-tests in TOXSTAT2006. For blood pressure (systolic, diastolic, and mean), respiratory, and ECG data, Bartlett’s test was first applied to assess homogeneity; if the data were homogeneous (*p* > 0.05), ANOVA (F-test) was conducted, followed by Dunnett’s parametric test for multiple comparisons if ANOVA results were significant (*p* ≤ 0.05). For non-homogeneous data (*p* ≤ 0.05), the Kruskal–Wallis test was used instead, with Dunnett’s non-parametric test applied for significant results (*p* ≤ 0.05); otherwise, the analysis was terminated.

## 3. Results

### 3.1. hERG Channel Inhibition Assay

**Figure 1:**
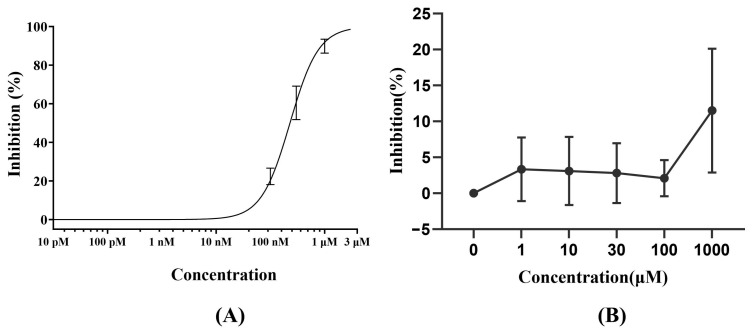
(**A**) The inhibitory effect of Cisapride on hERG current (*n* = 4). (**B**) The inhibitory effect of Deg-AZM on hERG current (*n* = 3).

To ensure the reliability of the experimental system, cisapride was employed as a positive control at concentrations of 0, 0.01, 0.1, 0.3, 1, and 3 μM. The IC_50_ value for hERG current inhibition (151.2 nM) was consistent with previously reported data (112.0 nM, Nanion) [25], confirming the stability and reproducibility of our assay. The positive control, cisapride, exhibited significant inhibition of hERG current with an IC_50_ of 151.2 nM (Figure 1A). In contrast, Deg-AZM demonstrated only weak inhibitory effects on hERG current. At 0.1 μM, Deg-AZM induced a maximal inhibition of 7.4%, while at 10 μM, the inhibition was 7.2%. Even at the highest tested concentration (1000 μM), the maximal inhibition reached only 21.3% (Figure 1B). As the maximum inhibition did not reach 50%, the IC_50_ value for Deg-AZM was estimated to be >1000 μM. The results indicated minimal hERG current inhibition, suggesting negligible hERG-blocking activity of Deg-AZM.

### 3.2. Cardiovascular and Respiratory Safety Pharmacology Study of Deg-AZM

#### 3.2.1. Blood Pressure of Beagle Dogs

**Figure 2:**
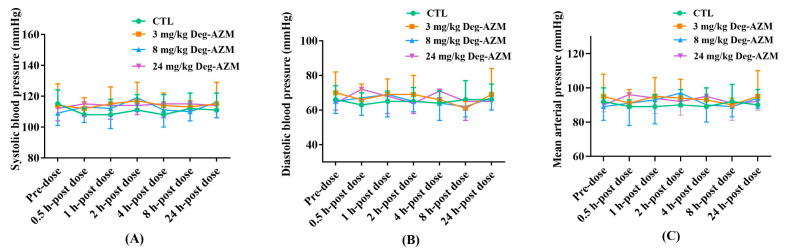
Changes in systolic blood pressure (**A**), diastolic blood pressure (**B**), and mean arterial pressure (**C**) in Beagle dogs at different time points after oral administration of Deg-AZM. Data were expressed as the means ± SD, *n* = 8.

Figure 2 illustrates the temporal pharmacodynamic profile of Deg-AZM on systolic blood pressure, diastolic blood pressure, and mean arterial pressure in Beagle dogs. Following oral administration at clinically relevant doses (3, 8, and 24 mg/kg), serial measurements of systolic blood pressure, diastolic blood pressure, and mean arterial pressure were obtained over a 24-h observation period. Quantitative analysis revealed that all three dose groups maintained hemodynamic parameters within physiologically normal ranges (systolic blood pressure: 108–119 mmHg; diastolic blood pressure: 61–72 mmHg; mean arterial pressure: 89–97 mmHg), with fluctuation patterns mirroring those of vehicle-treated controls. These findings collectively indicated that Deg-AZM administration, within the tested dose range, does not significantly perturb systemic arterial pressure homeostasis in canine models.

#### 3.2.2. Electrocardiographic Parameters in Beagle Dogs

**Figure 3:**
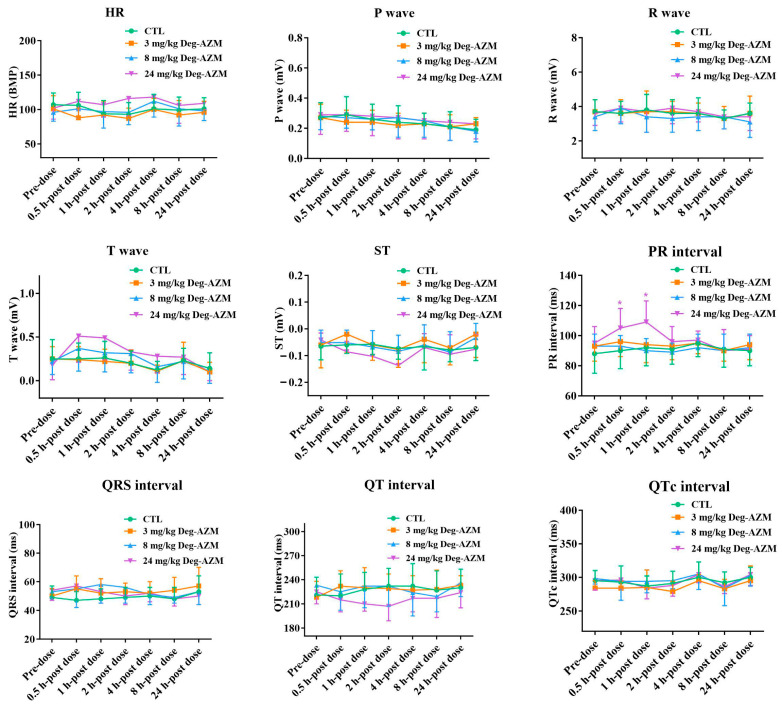
Changes in electrocardiographic parameters in Beagle dogs at different time points after oral administration of Deg-AZM. Data were expressed as the means ± SD, *n* = 8. * *p* < 0.05, vs. CTL group.

Electrocardiographic monitoring of Deg-AZM in Beagle dogs was displayed in Figure 3. The high-dose group (24 mg/kg) demonstrated statistically significant PR interval prolongation, with mean increases of 11% and 15% at 0.5 h and 1 h post-dosing, respectively, compared to vehicle controls. This transient atrioventricular conduction delay peaked at 1 h post-administration and returned to baseline levels by 4 h in all animals. The mid-dose (8 mg/kg) and low-dose (3 mg/kg) groups showed non-significant PR interval changes. No other parameters showed clinically relevant alterations versus the control group. These findings indicate that while high-dose Deg-AZM temporarily affects AV nodal conduction, it does not significantly impact other aspects of cardiac electrophysiology in this model.

#### 3.2.3. Respiratory Function in Beagle Dogs

**Figure 4:**
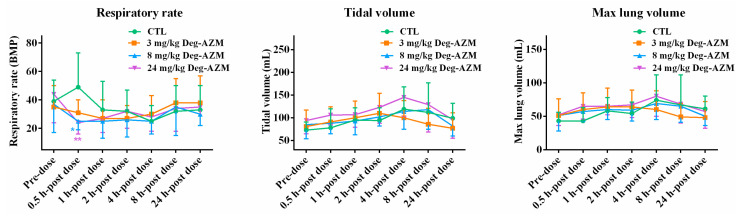
Changes in respiratory function (respiratory rate, tidal volume, maximal lung capacity) in Beagle dogs at different time points after oral administration of Deg-AZM. Data were expressed as the means ± SD, *n* = 8. * *p* < 0.05, ** *p* < 0.01, vs. CTL group.

Figure 4 presented the time-course effects of Deg-AZM on respiratory function in Beagle dogs, assessing respiratory rate, tidal volume, and maximal lung capacity. Compared with the vehicle control group, statistically significant decreases in respiratory rate were observed at 0.5 h post-dose in both the mid-dose (8 mg/kg) and high-dose (24 mg/kg) groups. This transient respiratory effect showed complete recovery by 2 h post-dose in all animals. Importantly, detailed examination of other pulmonary parameters demonstrated no significant alterations in tidal volume or maximal lung capacity across all time points.

### 3.3. Single-Dose Toxicity Study of Oral Deg-AZM Administration in Beagle Dogs

Throughout the study, vehicle control animals maintained good clinical condition with no mortality or moribundity. In the test article groups, no deaths occurred at 150 or 225 mg/kg doses. However, at 300 mg/kg, one animal died approximately 47 min post-dosing. Comprehensive assessments, including clinical signs (prone position, tremors, salivation, lethargy, deepened respiration, lateral recumbency, convulsions, vocalization, opisthotonos, restlessness, rigidity), electrocardiography (prolonged QT/QTc intervals and arrhythmias on dosing day), and histopathology (cardiac myocyte striation blurring), suggested acute cardiac ischemia/hypoxia induced by the test article.

#### 3.3.1. Toxicological Observations

Following oral administration of Deg-AZM (150 mg/kg) to Beagle dogs, clinical signs, including reduced spontaneous activity, vomiting, arched back posture, salivation, lethargy, increased respiratory depth, vocalization, and prone/squatting positions, were observed within 5 min post-dosing; tremors developed by 1 h post-administration, and at approximately 10 h after dosing, clinical signs had resolved in three of four animals while one animal continued to exhibit reduced spontaneous activity, lethargy, and tremors, which persisted in this affected animal when examined on the following day.

Following oral administration of Deg-AZM (225 mg/kg) to Beagle dogs, reduced spontaneous activity and prone positions were observed in one of four animals both pre-dose and at 5 min post-dosing; by 10 min after administration, animals developed tremors, vomiting, increased respiratory depth, arched back posture, squatting position, vocalization, prone/lateral recumbency, convulsions, restlessness, lethargy, opisthotonos, and tachypnea, with three of four animals showing complete recovery by approximately 10 h post-dose while one animal maintained reduced spontaneous activity and squatting position, though all animals returned to normal clinical status by the following day.

Following oral administration of Deg-AZM (300 mg/kg) to Beagle dogs, clinical signs including reduced spontaneous activity, squatting position, salivation, lethargy, arched back posture, vomiting, increased respiratory depth, vocalization, prone position, tremors, lateral recumbency, convulsions, opisthotonos, struggling, restlessness, and rigidity were observed within 5 min post-dosing; one animal (1/4) died at 47 min post-administration, and at approximately 6 h after dosing; remaining animals still exhibited reduced spontaneous activity, lethargy, arched back posture, squatting position, and tremors.

**Figure 5:**
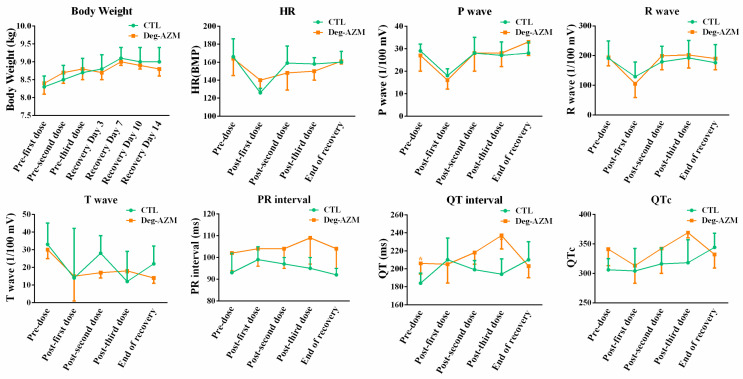
Body weight and ECG parameters in Beagle dogs following single oral administration of Deg-AZM. Data were expressed as the means ± SD, *n* = 4, * *p* < 0.05, vs. CTL group.

Throughout the study period, the Deg-AZM group demonstrated comparable body weight gain to the vehicle control group (Figure 5), with no abnormal findings observed in the comparative analysis. No feed residue was recorded for any animal over the entire course of the experiment.

#### 3.3.2. Electrocardiographic Examination

Electrocardiographic analysis of Beagle dogs administered Deg-AZM revealed dose-dependent QT/QTc interval prolongation 24 h post-second and third doses (Figure 5). Statistically significant QT prolongation was observed following the third administration compared to vehicle controls, suggesting a test article-related effect. All interval alterations normalized to vehicle control levels by the end of the 14-day recovery period.

#### 3.3.3. Hematological Examination

**Figure 6:**
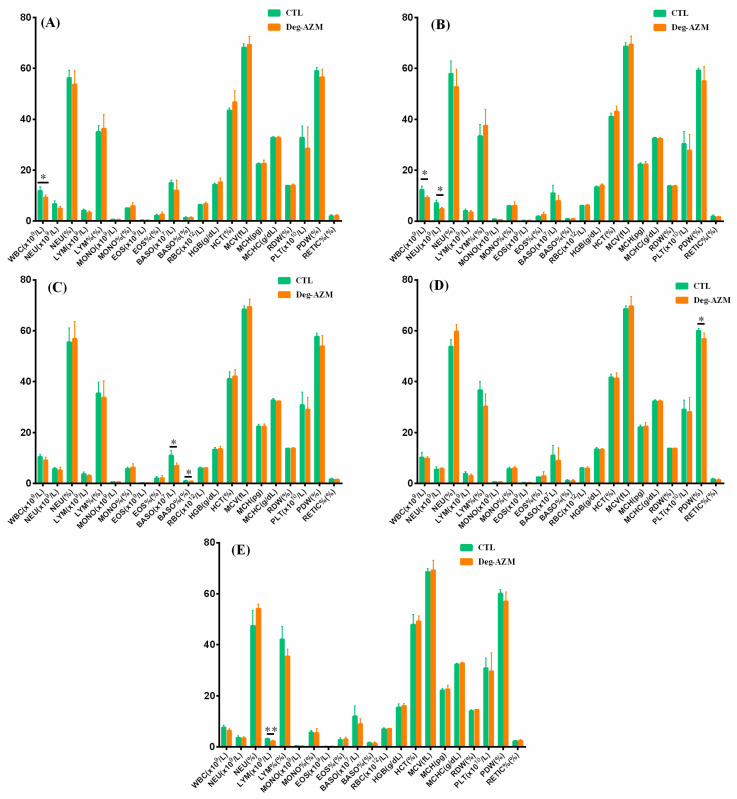
Hematological parameters in Beagle dogs following single oral administration of Deg-AZM at Pre-dose (**A**), Post-first dose (**B**), Post-second dose (**C**), Post-third dose (**D**), and End of recovery (**E**). Data were expressed as the means ± SD, *n* = 4. * *p* < 0.05, ** *p* < 0.01, vs. CTL group. (WBC, white blood cell count; NEU, neutrophil count; NEU%, neutrophil percentage; LYM, lymphocyte count; LYM%, lymphocyte percentage; MONO, monocyte count; MONO%, monocyte percentage; EOS, eosinophil count; EOS%, eosinophil percentage; BASO, basophil count; BASO%, basophil percentage; RBC, red blood cell count; HGB, hemoglobin concentration; HCT, hematocrit; MCV, mean corpuscular volume; MCH, mean corpuscular hemoglobin; MCHC, mean corpuscular hemoglobin concentration; RDW, red cell distribution width; PLT, platelet count; PDW, platelet distribution width; RETIC%, reticulocyte percentage).

Figure 6 presented the hematological results of Beagle dogs following oral administration of Deg-AZM. Compared to the CTL group, the Deg-AZM group exhibited significantly lower WBC values both pre-dosing and one day after the first administration (150 mg/kg), accompanied by reduced NEU levels post-first dose. Following the second dose (225 mg/kg), decreases in BASO and BASO% were observed, while the third dose (300 mg/kg) resulted in diminished PDW values, with persistently lower LYM levels detected at the recovery endpoint. The WBC reductions observed pre-dose and post-first administration—absent at higher doses—were attributed to inherent physiological variability, lacking toxicological relevance. Similarly, the transient decreases in NEU (post-first dose), BASO/BASO% (post-second dose), and LYM (recovery phase) showed no dose dependency and did not recur after the third administration, suggesting these changes were unrelated to the test article. Although PDW values were reduced post-third dose, PLT levels remained normal and PDW data demonstrated temporal consistency, indicating minimal clinical significance. Collectively, Deg-AZM administration across all tested doses induced no toxicologically relevant hematological abnormalities.

#### 3.3.4. Serum Biochemical Analysis

**Figure 7:**
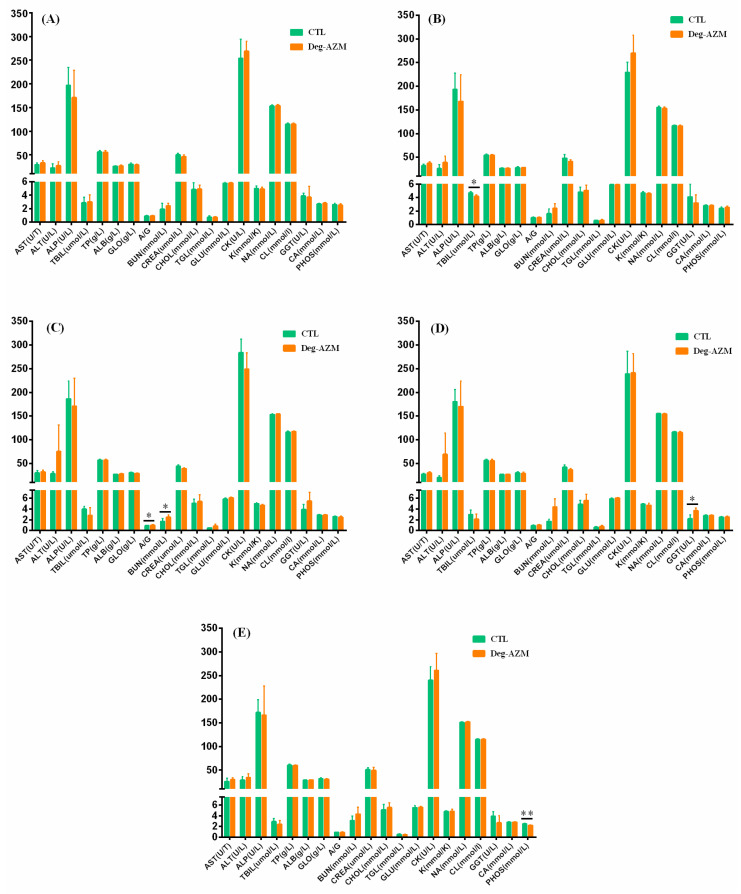
Serum biochemical parameters in Beagle dogs following single oral administration of Deg-AZM at Pre-dose (**A**), Post-first dose (**B**), Post-second dose (**C**), Post-third dose (**D**), and End of recovery (**E**). Data were expressed as the means ± SD, * *p* < 0.05, ** *p* < 0.01, vs. CTL group.

Figure 7 presented the serum biochemical results of Beagle dogs following oral administration of Deg-AZM. Compared to the CTL group, significantly lower TBIL levels were observed one day after the first administration (150 mg/kg), while increased A/G ratio and BUN levels were detected post-second dose (225 mg/kg). Following the third dose (300 mg/kg), elevated GGT levels were noted, with decreased PHOS values persisting through the recovery endpoint. The transient reduction in TBIL after the first administration was considered clinically insignificant. The elevated A/G and BUN levels showed temporal consistency with control group fluctuations, suggesting statistical rather than toxicological significance. Notably, the third-dose GGT elevation correlated with concurrent increases in ALT and ALP in individual animals, with consistently higher GGT levels observed after both the second and third administrations. Histopathological examination implicated the liver and heart as potential target organs, suggesting these biochemical alterations may represent Deg-AZM-induced effects.

#### 3.3.5. Toxicokinetic Analysis in Single-Dose Toxicity Study

**Figure 8:**
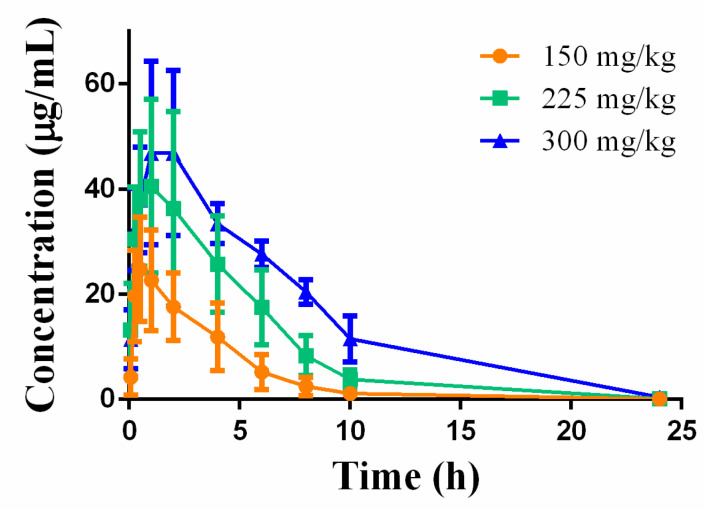
Mean plasma concentration-time curves of Deg-AZM in Beagle dogs following administration at different doses. Data were expressed as the means ± SD (*n* = 4).

The concentrations of Deg-AZM in plasma samples from Beagle dogs were quantified using HPLC-MS/MS to characterize the exposure–time relationship following drug administration, providing critical data for toxicological interpretation. After single oral doses of Deg-AZM (150, 225, and 300 mg/kg), the plasma concentration-time profiles were presented in Figure 8, with key pharmacokinetic parameters summarized in Table 1. Our results demonstrated dose-dependent increases in systemic exposure, with both AUC_0-t_ and C_max_ showing proportional escalation with higher doses. Across the three dose levels (150, 225, and 300 mg/kg; dose ratio 1:1.5:2.0), the relative ratios for AUC_0–t_ and C_max_ were 1:2.3:3.8 and 1:1.6:2.0, respectively, confirming a clear dose–exposure relationship.

To explore exposure–toxicity relationships, we compared systemic exposure (AUC_0–t_ and C_max_) with the incidence/severity of key clinical signs, ECG changes, and mortality. As shown in Table 1, mean AUC_0–t_ increased in an essentially dose-proportional manner from 100.72 μg/mL*h (150 mg/kg) to 386.34 μg/mL*h (300 mg/kg). The only mortality occurred at 300 mg/kg, where exposure reached ≥ 3.8-fold that of 150 mg/kg. QT/QTc prolongation ≥ 10% was first noted after the second dose (225 mg/kg; AUC_0–t_ ≈ 231.63 μg/mL*h), became statistically significant after the third dose (300 mg/kg), and coincided with C_max_ values ≥ 49 µg/mL. Clinical signs (reduced activity, tremor, vomiting) appeared at all doses but were transient and non-lethal at 150 and 225 mg kg^−1^. These data suggest that the threshold for life-threatening cardiotoxicity lies between 225 and 300 mg kg^−1^, corresponding to an AUC_0–t_ window of approximately 230–390 μg/mL*h and a C_max_ of ~40–50 µg/mL.

### 3.4. 28-Day Repeated Dose Oral Toxicity Study of Deg-AZM in Beagle Dogs

#### 3.4.1. Toxicological Observations

During the study period, animals in the CTL group exhibited normal general conditions without observable abnormalities, and no mortality or moribundity was observed in any group. The 5 mg/kg Deg-AZM group maintained good health status with no clinical signs. In the 15 mg/kg group during Week 1, clinical observations included the following: reduced spontaneous activity, prone/squatting posture, tremors, and conjunctival redness (1/10 animals); vomiting and soft stools (2/10); and loose stools (3/10). Loose stools persisted in 2/10 animals during Week 2, while no abnormalities were noted in Weeks 3–4. One of four recovery-phase animals exhibited loose stools in Week 1. The 50 mg/kg group showed frequent reductions in spontaneous activity, squatting, tremors, and vomiting during Week 1, with occasional observations of drowsiness, prone posture, soft/loose stools, conjunctival redness, and chills. Reduced activity remained common in Week 2 with occasional squatting and vomiting. By Week 3, sporadic activity reduction and vomiting persisted, while vomiting became the predominant observation in Week 4. During recovery monitoring, loose stools were observed in one of four 15 mg/kg animals during Week 1, while all other groups remained clinically unremarkable throughout this phase.

The Deg-AZM and CTL groups showed similar growth trajectories in body weight measurements, with no abnormalities noted in the comparative evaluation. Complete feed consumption was maintained across all treatment groups without exception.

**Figure 9:**
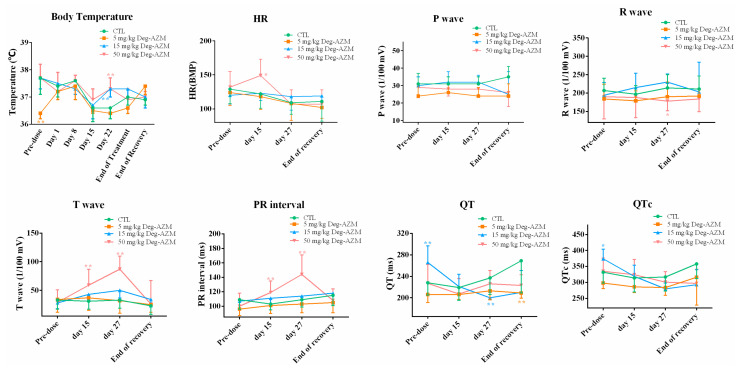
Body temperature and ECG parameters in Beagle dogs following 28-Day repeated dose oral administration of Deg-AZM. Data were expressed as the means ± SD, *n* = 4, * *p* < 0.05, ** *p* < 0.01, vs. CTL group.

Ophthalmic examinations revealed no abnormal findings across all treatment groups throughout the study. On Day 22, statistically significant elevations in body temperature were observed in the 15 mg/kg and 50 mg/kg Deg-AZM groups compared to the vehicle control (Figure 9). The absence of both temporal patterns and dose-dependency, coupled with the lack of observations in the high-dose group, suggests these transient temperature changes were unrelated to test article administration and devoid of toxicological relevance. All other scheduled temperature measurements remained within normal ranges for all groups.

#### 3.4.2. Electrocardiographic Examination

In the repeated oral dose toxicity study of Deg-AZM in Beagle dogs, no clinically significant drug-related alterations were observed in ECG parameters (Figure 9). The 15 mg/kg group exhibited prolongation of QT and QTc intervals prior to dosing. However, this effect was not observed following administration, suggesting it was attributable to normal inter-animal variation without toxicological significance. After the 15th administration, the 50 mg/kg group demonstrated increased HR, elevated T-wave amplitude, and prolonged PR interval. These changes persisted through the 27th administration, with the 50 mg/kg group maintaining T-wave elevation and PR prolongation, some of which showed statistically significant differences compared to the CTL group. During the recovery period, the 5 mg/kg group displayed a slight shortening of QT interval (<2% change, correlated with CTL control fluctuations), while all other ECG parameters returned to normal ranges across treatment groups. Further analysis revealed that the ECG alterations in the 50 mg/kg group (increased HR, T-wave elevation, and PR prolongation) exhibited time-dependent progression and were associated with histopathological findings of myocardial vacuolation and scattered eosinophilic degeneration, suggesting these ECG changes may reflect drug-related cardiac effects that were reversible upon treatment withdrawal. In conclusion, with the exception of the reversible cardiac effects observed in the 50 mg/kg group, no ECG abnormalities of clear toxicological significance were detected in the remaining dose groups.

#### 3.4.3. Hematological Examination and Coagulation Tests

Hematological and coagulation test results from Beagle dogs receiving repeated oral doses of Deg-AZM are presented in Table 2. No abnormalities were observed in hematological or coagulation parameters in the 5 mg/kg group throughout the dosing period. Compared to the CTL group, animals in the 50 mg/kg group showed a significant decrease in RETIC% values after both 15 and 27 days of continuous administration, while all other parameters remained normal. Following the recovery period, all parameters returned to normal ranges in all groups. A comprehensive analysis incorporating temporal changes in RETIC% values and other erythroid-related hematological parameters across different treatment groups suggested that the observed reduction in RETIC% alone lacked clear toxicological significance. Importantly, all three dose groups (5, 15, and 50 mg/kg) demonstrated normal hematological and coagulation profiles throughout the study, indicating no treatment-related effects on these parameters at the tested doses. The transient RETIC% reduction observed specifically in the high-dose group (50 mg/kg) was considered an isolated finding without pathological correlation, as it was not accompanied by changes in other erythroid parameters (including RBC count, hemoglobin, and hematocrit) and showed complete reversibility during recovery. These findings collectively support the absence of clinically meaningful hematological or coagulation alterations following Deg-AZM administration at the studied dose levels.

#### 3.4.4. Serum Biochemical Analysis

The serum biochemical parameters measured in Beagle dogs following repeated oral administration of Deg-AZM are summarized in Table 3. Following comparative analysis with the CTL group, the 5 mg/kg group showed significantly decreased serum CK levels and elevated NA values after 15 days of continuous administration, while the 50 mg/kg group exhibited significantly reduced ALB and A/G ratios. At day 27, the 5 mg/kg group demonstrated significantly lower GGT levels and the 15 mg/kg group showed decreased PHOS values, with all other serum biochemical parameters remaining normal during other observation periods. Pretreatment elevations in GLO and reductions in A/G ratio observed in the 5 mg/kg group were attributed to lower baseline values in controls, as these parameters normalized post-dosing and were considered transient physiological variations. The clinically insignificant CK reduction in the 5 mg/kg group at day 15 and the transient NA elevation (which disappeared by study end) showed no temporal correlation with treatment. Similarly, the day-27 GGT decrease in the 5 mg/kg group was deemed clinically irrelevant. The ALB and A/G reductions in the 50 mg/kg group at day 15 lacked a dose–response relationship as they were not observed at higher doses or at study termination. The terminal PHOS reduction in the 15 mg/kg group showed no dose-dependency. Individual data analysis revealed that pretreatment AST/ALT elevations in some 15 mg/kg animals correlated with hepatic granulomas found histopathologically, indicating spontaneous liver lesions rather than test article-related effects. In conclusion, no Deg-AZM-related alterations were observed in serum biochemical parameters across all dose groups.

#### 3.4.5. Toxicokinetic Analysis in 28-Day Repeated Dose Oral Toxicity Study

The concentrations of Deg-AZM in plasma samples from Beagle dogs were quantified using HPLC-MS/MS to characterize the exposure–time relationship following drug administration, providing critical data for toxicological interpretation. After repeated oral administration of Deg-AZM in Beagle dogs (5, 15, and 50 mg/kg), the key pharmacokinetic parameters are summarized in Table 4. Deg-AZM exposure showed no gender-related differences across the 5–50 mg/kg dose range. The three dose groups (5, 15, and 50 mg/kg; ratio 1:3:10) demonstrated dose-proportional pharmacokinetics for both AUC_0-t_ and C_max_. Following initial administration, male Beagles exhibited AUC_0-t_ and C_max_ ratios of 1:2:4.8 and 1:1.6:4.3, respectively, while females showed ratios of 1:2.4:8.4 (AUC_0-t_) and 1:2:4.7 (C_max_). At terminal dosing, these ratios were 1:2.2:5.5 (AUC_0-t_) and 1:2.3:5.7 (C_max_) in males, versus 1:2:7.6 (AUC_0-t_) and 1:1.8:5.8 (C_max_) in females. After 28 days of repeated administration, accumulation factors (terminal/initial AUC_0-t_)) were 1.38, 1.4, and 1.5 for the 5, 15, and 50 mg/kg groups, respectively, indicating minimal accumulation potential.

To define exposure thresholds for repeated-dose effects, we compared steady-state AUC_0–t_ (Day 28) with the onset and severity of cardiac findings. The low dose (5 mg/kg/day) produced an AUC_0–t_ of 7.6–8.0 μg/mL*h (males and females, respectively) and was devoid of any ECG or histopathological changes. The mid dose (15 mg/kg/day) led to an AUC_0–t_ of 16–17 μg/mL*h; transient soft stool and reduced activity occurred during Week 1, but no cardiac alterations were observed. The high dose (50 mg/kg/day) achieved an AUC_0–t_ of 42–61 μg/mL*h and was associated with persistent PR-interval prolongation, elevated T-wave amplitude, and reversible myocardial vacuolation.

#### 3.4.6. Pathological Examination

**Figure 10:**
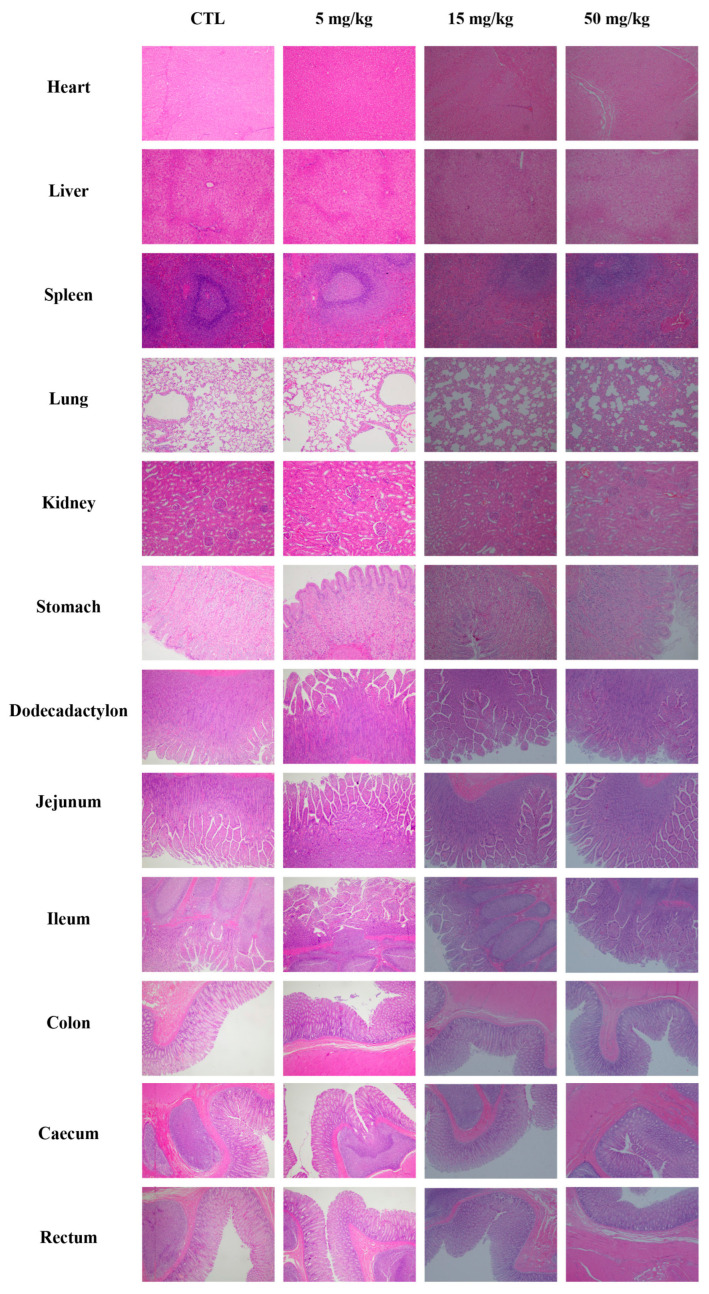
Histopathological examination in Beagle dogs following 28-day repeated-dose oral administration of Deg-AZM.

Histopathological examination (Figure 10) revealed no test article-related findings in the 5 and 15 mg/kg groups at both terminal sacrifices. The 50 mg/kg group exhibited minimal myocardial vacuolar degeneration and scattered eosinophilic changes in individual cardiomyocytes. All observed histopathological changes were fully reversible after the recovery period, as no related findings were detected in any dose group at study conclusion. These results demonstrated that the myocardial findings at the high dose (50 mg/kg) were transient and reversible upon treatment cessation.

## 4. Discussion

The comprehensive preclinical safety evaluation of Deg-AZM, a novel class I transgelin agonist for STC, was established. It demonstrated a favorable safety profile, characterized by minimal cardiac ion channel risk and manageable, reversible toxicity. Inhibition of the hERG potassium channel is a critical safety concern for new chemical entities, as it is associated with potentially life-threatening arrhythmias such as Torsades de Pointes [21,22]. Importantly, while the introduction highlights hERG inhibition as a general safety concern for New Chemical Entities, our results definitively showed that Deg-AZM is not a potent inhibitor of the hERG channel (IC_50_ > 1000 μM), effectively mitigating this specific cardiac risk and distinguishing its off-target safety profile from its primary therapeutic mechanism of action on Transgelin. In our study, Deg-AZM demonstrated minimal hERG channel inhibition, with a maximal inhibition of only 21.3% at the highest tested concentration of 1000 μM. This was in stark contrast with the positive control cisapride, which exhibited an IC_50_ value of 151.2 nM. The negligible hERG-blocking activity of Deg-AZM suggested a low risk of cardiac toxicity, which was a promising feature for its clinical development.

The cardiovascular and respiratory safety pharmacology studies in conscious Beagle dogs revealed that Deg-AZM had minimal effects on vital physiological functions at clinically relevant doses. Although a transient prolongation of the PR interval was observed at the high dose (24 mg/kg), this effect was short-lived and resolved within 4 h post-dosing. No significant changes were observed in other electrocardiographic parameters, indicating that Deg-AZM does not significantly impact cardiac electrophysiology. Similarly, the transient decrease in respiratory rate at the mid and high doses did not affect other respiratory parameters and was fully reversible within 2 h. These findings suggest that Deg-AZM is unlikely to cause clinically relevant cardiovascular or respiratory adverse effects at therapeutic doses. The single-dose toxicity study demonstrated that Deg-AZM exhibited dose-dependent effects, with one death occurring at the highest dose of 300 mg/kg. The observed clinical signs, including reduced spontaneous activity, tremors, and vomiting, were consistent with acute cardiac ischemia/hypoxia, as supported by electrocardiographic and histopathological findings. However, these severe effects were not observed at lower doses (150 and 225 mg/kg), indicating that the toxicity profile of Deg-AZM was dose-dependent. The 28-day repeated-dose toxicity study further confirmed the safety profile of Deg-AZM. At the lowest dose of 5 mg/kg, no significant clinical signs or toxicological effects were observed. However, at higher doses (15 and 50 mg/kg), transient clinical symptoms, such as reduced activity and vomiting, were noted, particularly in the first week of dosing. These symptoms were reversible upon cessation of treatment, indicating that Deg-AZM does not cause persistent adverse effects. Notably, the electrocardiographic changes observed in the 50 mg/kg group, such as PR interval prolongation and T-wave elevation, were associated with histopathological findings of myocardial vacuolar degeneration. These changes were fully reversible during the recovery period, suggesting that the cardiac effects of Deg-AZM are reversible and dose-dependent. This histopathological finding, characterized by minimal vacuolar degeneration, and scattered eosinophilic changes in cardiomyocytes, demonstrated complete reversibility after the 28-day recovery period—a key indicator of low chronic toxicity risk.

Current pharmacological treatments for STC primarily include bulk-forming laxatives, osmotic laxatives, stimulant laxatives, guanylate cyclase-C (GC-C) agonists, highly selective 5-Hydroxytryptamine (5-HT) receptor agonists, chloride channel activators (ClC), and probiotics [26]. Among these, prucalopride, lubiprostone, and linaclotide could alleviate constipation by enhancing intestinal motility. Prucalopride is a selective high-affinity serotonin 5-HT_4_ receptor agonist that enhances colonic motility by stimulating enteric cholinergic and nitrergic neurons to accelerate gut transit [27,28]. Lubiprostone is a locally acting ClC-2 chloride channel activator that promotes intestinal fluid secretion by activating apical ClC-2 channels in epithelial cells, thereby softening stool and stimulating peristalsis [29,30]. Linaclotide is a GC-C agonist that binds to and activates GC-C on the intestinal epithelial surface, stimulating the production of cyclic guanosine monophosphate (cGMP). The increased cGMP triggers a signaling cascade, activating the cystic fibrosis transmembrane conductance regulator. This activation promotes the secretion of chloride and bicarbonate into the intestinal lumen, thereby increasing intestinal fluid secretion, accelerating gastrointestinal transit, and facilitating bowel movements [31]. Using established atropine-induced mouse constipation models, we evaluated the therapeutic efficacy of Deg-AZM with prucalopride as the positive control drug. The results displayed in Figure S1 in Supplementary Materials demonstrated that oral administration of Deg-AZM significantly increased fecal dry weight, wet weight, and feces number compared to the Model group, while markedly elevating the carbon powder propulsion rate. Notably, animals receiving Deg-AZM at 10 mg/kg and 15 mg/kg exhibited higher propulsion rates than the positive control group. Compared to these marketed drugs for STC currently, Deg-AZM represents a mechanistically novel approach by directly activating a transgelin-dependent contractile pathway in gastrointestinal smooth muscle cells. This distinct mechanism bypasses both serotonergic neuronal signaling and epithelial chloride secretion. Traditional 5-HT_4_ agonists may off-target inhibit the cardiac hERG potassium channel (IKr current), potentially leading to QT interval prolongation and significantly increasing the risk of Torsades de Pointes. The withdrawal of cisapride due to fatal arrhythmias has clearly highlighted its clinical severity [32,33]. In contrast, Deg-AZM targets transgelin and does not rely on the 5-HT_4_ receptor. Although Deg-AZM does not carry the specific cardiovascular risks associated with 5-HT_4_ agonism, our studies have revealed that it exhibits certain dose-dependent cardiotoxic effects, including PR interval prolongation, elevated T-waves, and reversible myocardial vacuolation at high dosage (50 mg/kg in the 28-day study and 300 mg/kg in the single-dose study), as well as a single incidence of mortality in acute toxicity studies. These findings suggested that Deg-AZM may have potential cardiovascular effects under conditions of extremely high exposure. It is important to acknowledge these findings and place them in the context of clinical risk. For clinical trial design, the No Observed Adverse Effect Level (NOAEL) of 5 mg/kg established in the 28-day safety study of dog could serve as the basis for Human Equivalent Dose (HED) calculation using body surface area normalization (HED = Animal NOAEL × [Animal Km/Human Km]). Applying the standard Km values (dog: 20; human: 37) [34], this yielded an HED of 2.70 mg/kg (5 × 20/37). For a 60 kg human, the absolute HED is 162 mg. When determining the first-in-human starting dose, a 20-fold safety factor was applied to the HED to establish a Maximum Recommended Starting Dose (MRSD) of 8.1 mg. The predefined maximum clinical dose remains below this HED value, with rigorous safety monitoring (including comprehensive clinical parameters and pharmacokinetic exposure assessments) implemented throughout the trial to ensure participant safety. Mechanistically, Deg-AZM exhibited a favorable preclinical profile suggesting low clinical safety risk. A potential mechanistic rationale for the observed myocardial vacuolation may involve lysosomal phospholipidosis, a class effect associated with some cationic amphiphilic drugs (CADs), including certain macrolides [35]. As a macrolide derivative, Deg-AZM’s structure suggests it could possess CAD-like properties, potentially leading to phospholipid accumulation in lysosomes of cardiomyocytes at high concentrations. This phenomenon is often reversible and consistent with the transient nature of our histopathological findings. However, further investigative studies would be required to confirm this specific mechanism.

Deg-AZM ameliorates chronic constipation by functioning as a Transgelin agonist that biomechanically promotes actin polymerization, thereby enhancing stress fiber bundle formation in intestinal smooth muscle cells. This mechanism augments cellular contractility and intestinal peristalsis, ultimately resolving constipation symptoms. The compound demonstrates gastrointestinal tract-specific action with minimal off-target effects. In preclinical canine toxicology studies, adverse findings occurred exclusively in mid- and high-dose groups, with all toxicities proving fully reversible upon treatment cessation. As the first Transgelin-targeting agent developed for treatment of STC, Deg-AZM represented a novel therapeutic strategy with significant potential to address unmet clinical needs in patients exhibiting poor response or intolerance to conventional therapies.

## 5. Conclusions

This study revealed the comprehensive preclinical safety profile of Deg-AZM, a novel clinical-stage Transgelin agonist. Overall, Deg-AZM exhibited a favorable preclinical safety profile. While Deg-AZM exhibited some cardiotoxic effects at high dosage, no significant hERG channel inhibition was observed. Importantly, cardiotoxicity of Deg-AZM in dogs was reversible (myocardial vacuolation resolved post-recovery). The compound showed dose-proportional systemic exposure without notable accumulation. Clinical safety data could not be obtained in the preclinical stage; this integrated safety assessment could undoubtedly be an important reference for clinical trial design, effectively de-risking phase I human studies.

## Figures and Tables

**Table 1:** Pharmacokinetic parameters of Deg-AZM in plasma of Beagle dogs. Data were expressed as the means ± SD (*n* = 4).

Parameters	150 mg/kg	225 mg/kg	300 mg/kg
AUC_0-t_ (μg/mL*h)	100.72 ± 43.51	231.63 ± 84.21	386.34 ± 43.85
C_max_ (μg/mL)	25.20 ± 9.46	41.53 ± 17.24	49.27 ± 19.89
T_1/2_ (h)	1.74 ± 0.23	1.98 ± 0.32	2.60 ± 0.94
T_max_ (h)	0.63 ± 0.25	1.38 ± 0.75	1.67 ± 0.58

**Table 2:** Hematological and coagulation parameters in Beagle dogs receiving repeated oral doses of Deg-AZM.

Parameters	Pre-Dose				Day 15				Day 27				End of Recovery			
	CTL	5 mg/kg	15 mg/kg	50 mg/kg	CTL	5 mg/kg	15 mg/kg	50 mg/kg	CTL	5 mg/kg	15 mg/kg	50 mg/kg	CTL	5 mg/kg	15 mg/kg	50 mg/kg
WBC (×10^9^/L)	10.7 ± 1.8	8.8 ± 2.4	10.1 ± 1.7	9.4 ± 1.4	8.4 ± 1.4	11.1 ± 3.5	8.7 ± 1.4	8.7 ± 1.1	7.9 ± 1.1	10.4 ± 2.1	8.5 ± 2.3	7.7 ± 1.5	11.3 ± 1.5	10.3 ± 1.2	9.4 ± 0.7	9.5 ± 1.4
NEU% (%)	5.6 ± 1.2	4.7 ± 1.3	6.2 ± 1.1	5.5 ± 1.0	4.6 ± 1.0	6.5 ± 3.0	5.0 ± 1.0	4.8 ± 0.9	4.5 ± 0.8	5.8 ± 1.7	4.6 ± 1.6	4.1 ± 1.0	7.0 ± 1.0	6.0 ± 1.2	5.4 ± 0.4	5.2 ± 0.9
NEU% (%)	52.4 ± 5.8	52.8 ± 4.2	61.4 ± 3.3	58.4 ± 3.8	54.9 ± 5.0	57.2 ± 6.7	56.7 ± 4.9	54.8 ± 4.5	56.2 ± 4.4	56.9 ± 6.4	54.0 ± 5.8	53.4 ± 3.8	62.2 ± 3.3	58.1 ± 5.9	57.2 ± 1.5	54.3 ± 5.9
LYM (×10^9^/L)	3.6 ± 0.9	3.0 ± 1.0	3.0 ± 0.5	3.0 ± 0.4	3.0 ± 0.6	3.2 ± 0.5	3.0 ± 0.5	3.1 ± 0.4	2.7 ± 0.5	3.1 ± 0.6	3.0 ± 0.7	2.8 ± 0.6	3.0 ± 0.5	2.8 ± 0.1	3.1 ± 0.2	3.7 ± 0.7
LYM% (%)	33.5 ± 6.8	34.1 ± 4.2	29.4 ± 3.6	32.6 ± 4.4	36.1 ± 4.0	30.2 ± 6.3	34.1 ± 4.2	35.4 ± 4.8	34.2 ± 4.0	31.7 ± 5.5	36.0 ± 5.4	36.4 ± 3.8	26.3 ± 3.7	27.9 ± 3.5	33.4 ± 2.4	38.3 ± 4.9
MONO (×10^9^/L)	0.7 ± 0.2	0.6 ± 0.2	0.6 ± 0.2	0.6 ± 0.2	0.5 ± 0.1	0.8 ± 0.3	0.5 ± 0.1	0.6 ± 0.1	0.5 ± 0.1	0.6 ± 0.1	0.5 ± 0.2	0.5 ± 0.2	0.9 ± 0.2	0.8 ± 0.0	0.5 ± 0.1	0.4 ± 0.1
MONO% (%)	6.9 ± 1.6	6.8 ± 1.1	5.7 ± 1.0	6.0 ± 1.2	5.9 ± 1.8	7.3 ± 1.6	6.1 ± 1.2	6.4 ± 1.2	6.1 ± 1.4	6.1 ± 0.8	5.4 ± 1.5	6.0 ± 1.2	7.6 ± 1.3	7.6 ± 0.6	5.7 ± 1.1	4.0 ± 0.5
EOS (×10^9^/L)	0.5 ± 0.5	0.4 ± 0.2	0.3 ± 0.3	0.2 ± 0.1	0.2 ± 0.1	0.4 ± 0.2	0.2 ± 0.1	0.2 ± 0.1	0.2 ± 0.1	0.3 ± 0.2	0.3 ± 0.2	0.2 ± 0.2	0.3 ± 0.2	0.5 ± 0.2	0.2 ± 0.1	0.2 ± 0.10
EOS% (%)	4.9 ± 3.9	4.2 ± 2.5	2.5 ± 1.7	1.9 ± 1.0	2.2 ± 0.8	3.3 ± 1.5	2.1 ± 0.9	2.4 ± 1.2	2.5 ± 1.1	3.3 ± 1.8	3.1 ± 1.9	3.2 ± 2.5	2.9 ± 1.5	5.1 ± 2.1	2.5 ± 1.2	2.3 ± 1.0
BASO (×10^9^/L)	0.1 ± 0.0	0.1 ± 0.0	0.1 ± 0.1	0.1 ± 0.0	0.1 ± 0.0	0.1 ± 0.0	0.1 ± 0.0	0.1 ± 0.0	0.1 ± 0.0	0.1 ± 0.0	0.1 ± 0.1	0.1 ± 0.0	0.1 ± 0.1	0.1 ± 0.0	0.1 ± 0.0	0.1 ± 0.0
BASO% (%)	0.7 ± 0.3	0.7 ± 0.3	0.7 ± 0.4	0.7 ± 0.3	0.7 ± 0.2	0.6 ± 0.2	0.7 ± 0.2	0.6 ± 0.3	0.6 ± 0.2	0.9 ± 0.3	1.0 ± 0.8	0.7 ± 0.3	0.5 ± 0.1	0.6 ± 0.2	0.9 ± 0.4	0.7 ± 0.2
RBC (×10^12^/L)	6.3 ± 0.4	5.6 ± 1.4	6.3 ± 0.3	6.6 ± 0.6	6.5 ± 0.5	6.3 ± 0.5	6.6 ± 0.4	6.3 ± 0.6	6.7 ± 0.4	6.4 ± 0.7	6.6 ± 0.4	6.4 ± 0.4	6.7 ± 0.3	6.6 ± 0.1	6.9 ± 0.3	6.8 ± 0.3
HGB (g/dL)	15.3 ± 1.2	15.1 ± 0.9	13.8 ± 0.7	14.5 ± 1.2	14.5 ± 0.8	14.7 ± 1.2	14.4 ± 0.7	14.0 ± 1.3	14.9 ± 0.7	14.8 ± 1.5	14.5 ± 0.8	14.2 ± 1.1	15.3 ± 1.1	15.4 ± 0.2	15.7 ± 1.1	15.3 ± 1.5
HCT (%)	46.8 ± 3.4	40.7 ± 11.3	42.6 ± 1.9	44.2 ± 3.6	44.5 ± 2.6	45.0 ± 3.5	44.3 ± 2.5	42.6 ± 4.0	45.0 ± 2.1	45.5 ± 4.8	43.7 ± 2.3	42.8 ± 3.1	47.7 ± 3.0	47.3 ± 0.6	47.4 ± 2.5	45.1 ± 4.5
MCV (fL)	74.0 ± 1.5	72.4 ± 3.6	67.7 ± 2.0	67.4 ± 2.0	68.6 ± 1.7	71.8 ± 2.0	67.8 ± 1.7	67.4 ± 1.6	67.7 ± 1.4	70.9 ± 2.0	66.7 ± 2.0	66.6 ± 1.7	71.2 ± 1.5	71.4 ± 1.3	69.0 ± 1.1	66.3 ± 0.6
MCH (pg)	24.2 ± 0.6	29.7 ± 11.6	21.8 ± 0.7	22.0 ± 0.7	22.4 ± 0.5	23.5 ± 0.7	22.0 ± 0.6	22.2 ± 0.8	22.4 ± 0.5	23.1 ± 0.5	13.4 ± 0.5	22.1 ± 0.7	22.8 ± 0.7	23.2 ± 0.5	22.8 ± 0.5	22.5 ± 0.3
MCHC (g/dL)	32.7 ± 0.8	41.7 ± 18.8	32.3 ± 0.5	32.7 ± 0.3	32.6 ± 0.6	32.7 ± 0.5	32.5 ± 0.4	32.9 ± 0.5	33.2 ± 0.3	32.6 ± 0.5	22.1 ± 0.7	33.2 ± 0.5	32.1 ± 0.5	32.5 ± 0.2	33.1 ± 0.6	33.9 ± 0.4
RDW (%)	13.1 ± 0.4	14.8 ± 3.4	13.5 ± 0.5	13.5 ± 0.6	13.9 ± 0.9	13.3 ± 0.3	13.5 ± 0.5	13.7 ± 0.8	13.6 ± 0.9	13.1 ± 0.3	33.1 ± 0.3	13.7 ± 0.8	13.0 ± 0.2	13.0 ± 0.3	13.6 ± 0.3	14.3 ± 0.7
PLT (×10^9^/L)	331 ± 75	697 ± 721	388 ± 35	444 ± 46	335 ± 39	293 ± 63	347 ± 36	402 ± 92	324 ± 38	283 ± 52	13.4 ± 0.5	365 ± 102	256 ± 67	230 ± 40	305 ± 43	380 ± 99
PDW (%)	50.2 ± 5.0	45.0 ± 8.8	57.4 ± 3.0	56.4 ± 4.8	53.8 ± 2.5	51.8 ± 3.5	53.7 ± 3.7	54.8 ± 3.5	54.0 ± 3.6	53.1 ± 2.0	342 ± 26	54.1 ± 5.4	49.4 ± 3.5	52.4 ± 4.9	56.5 ± 1.9	55.3 ± 2.4
RETIC (%)	1.2 ± 0.4	1.7 ± 0.5 *	2.2 ± 0.6	1.7 ± 0.5	2.1 ± 0.8	1.7 ± 0.4	2.2 ± 0.5	1.4 ± 0.4 **	1.8 ± 0.8	1.4 ± 0.3	56.5 ± 3.8	1.2 ± 0.4 **	1.2 ± 0.4	1.6 ± 0.4	2.0 ± 0.2	1.8 ± 0.4
PT (s)	75.0 ± 25.3	105.6 ± 34.0 *	8.2 ± 0.7	8.6 ± 1.2	8.1 ± 0.6	7.2 ± 0.4	8.0 ± 0.6	8.4 ± 1.1	8.5 ± 0.7	7.5 ± 0.4	1.57 ± 0.39	8.5 ± 1.0	7.5 ± 0.7	7.5 ± 0.2	7.6 ± 0.3	8.2 ± 0.7
APTT (s)	6.2 ± 0.5	6.1 ± 0.3	13.1 ± 1.4	13.6 ± 1.8	14.7 ± 1.1	16.0 ± 1.9	14.4 ± 1.1	14.3 ± 2.6	15.1 ± 2.0	16.9 ± 2.9	8.1 ± 0.8	13.8 ± 2.0	18.2 ± 1.2	19.3 ± 2.3	16.3 ± 2.6	16.9 ± 1.4
FIB (mg/dL)	15.0 ± 2.7	14.7 ± 2.7	257 ± 43	261 ± 41	239 ± 36	276 ± 78	262 ± 43	290 ± 106	231 ± 26	294 ± 88	14.3 ± 2.0	225 ± 49	265 ± 28	307 ± 83	217 ± 26	207 ± 39
TT (s)	240 ± 72	-	9.9 ± 0.4	9.9 ± 0.4	11.5 ± 1.5	9.2 ± 0.3	11.5 ± 1.8	11.2 ± 1.3	10.6 ± 0.4	9.1 ± 0.3	251 ± 40	10.6 ± 0.6	10.4 ± 0.3	10.0 ± 0.3	10.6 ± 0.6	11.3 ± 0.9

Data were expressed as mean ± SD (*n* = 4). * *p* < 0.05, ** *p* < 0.01, vs. CTL group.

**Table 3:** The serum biochemical parameters in Beagle dogs receiving repeated oral doses of Deg-AZM.

Parameters	Pre-Dose				Day 15				Day 27				End of Recovery			
	CTL	5 mg/kg	15 mg/kg	50 mg/kg	CTL	5 mg/kg	15 mg/kg	50 mg/kg	CTL	5 mg/kg	15 mg/kg	50 mg/kg	CTL	5 mg/kg	15 mg/kg	50 mg/kg
AST (U/L)	37 ± 9	22 ± 4	43 ± 28	35 ± 7	30 ± 6	26 ± 5	32 ± 7	33 ± 5	31 ± 4	26 ± 5	71 ± 134	30 ± 4	29 ± 11	23 ± 5	31 ± 10	34 ± 4
ALT (U/L)	33 ± 13	34 ± 15	85 ± 196	33 ± 13	28 ± 11	29 ± 9	26 ± 8	30 ± 9	31 ± 9	31 ± 12	156 ± 413	27 ± 8	42 ± 17	36 ± 8	23 ± 9	27 ± 13
ALP (U/L)	180 ± 41	144 ± 27	165 ± 41	154 ± 32	187 ± 35	160 ± 33	176 ± 40	162 ± 33	177 ± 37	161 ± 23	164 ± 33	160 ± 33	192 ± 43	150 ± 25	182 ± 26	142 ± 11
TBIL (µmol/L)	3.0 ± 0.9	2.7 ± 0.2	3.1 ± 0.9	3.4 ± 0.7	2.7 ± 0.5	1.8 ± 0.5	3.5 ± 0.4	2.9 ± 0.7	3.5 ± 0.8	2.6 ± 0.4	3.5 ± 1.1	3.5 ± 0.9	3.7 ± 0.5	2.5 ± 0.7	3.7 ± 2.3	3.4 ± 1.6
TP (g/L)	59.6 ± 3.7	55.0 ± 3.2	59.4 ± 1.8	59.5 ± 2.1	58.9 ± 2.8	57.9 ± 2.9	59.0 ± 1.8	58.4 ± 2.1	58.9 ± 3.3	57.4 ± 2.7	59.8 ± 3.1	58.0 ± 1.4	60.1 ± 3.6	56.9 ± 3.9	60.6 ± 2.6	56.6 ± 2.3
ALB (g/L)	27.6 ± 2.0	27.8 ± 2.0	27.1 ± 0.9	27.5 ± 1.3	27.4 ± 1.5	29.3 ± 2.0	26.4 ± 1.0	25.6 ± 1.3 **	27.5 ± 1.9	28.5 ± 2.3	26.9 ± 1.6	26.6 ± 1.2	28.3 ± 1.5	27.6 ± 3.0	27.7 ± 1.8	26.5 ± 0.5
GLO (g/L)	32.0 ± 2.3	27.2 ± 3.9 **	32.4 ± 2.0	32.0 ± 2.4	31.5 ± 2.1	28.7 ± 4.3	32.7 ± 1.3	32.8 ± 2.2	31.4 ± 2.5	29.0 ± 4.0	32.9 ± 2.1	31.5 ± 1.0	31.7 ± 2.2	29.3 ± 6.3	32.9 ± 1.9	30.1 ± 2.0
A/G	0.9 ± 0.1	1.0 ± 0.2 *	0.8 ± 0.1	0.9 ± 0.1	0.9 ± 0.1	1.0 ± 0.2	0.8 ± 0.0	0.8 ± 0.1 *	0.9 ± 0.1	1.0 ± 0.2	0.8 ± 0.0	0.9 ± 0.1	0.9 ± 0.0	1.0 ± 0.3	0.9 ± 0.1	0.9 ± 0.1
BUN (mmol/L)	2.2 ± 0.6	3.1 ± 0.6	1.9 ± 0.4	2.3 ± 0.5	3.0 ± 0.5	3.3 ± 1.1	2.7 ± 0.9	3.4 ± 0.7	2.9 ± 1.0	3.5 ± 0.9	2.8 ± 1.3	2.9 ± 0.7	3.1 ± 0.6	4.7 ± 1.5	2.4 ± 0.5	3.3 ± 0.5
CREA (µmol/L)	41 ± 12	61 ± 6	37 ± 7	44 ± 10	38 ± 7	60 ± 7	34 ± 8	37 ± 10	53 ± 6	63 ± 6	50 ± 9	59 ± 8	48 ± 8	61 ± 7	49 ± 8	53 ± 12
CHOL (mmol/L)	4.3 ± 0.7	5.7 ± 0.7	4.3 ± 0.9	4.7 ± 0.8	4.7 ± 0.8	5.7 ± 1.1	4.6 ± 0.9	5.4 ± 1.2	4.7 ± 0.9	5.4 ± 1.2	4.9 ± 1.4	5.3 ± 0.9	5.0 ± 0.1	5.2 ± 0.9	4.6 ± 0.4	4.4 ± 1.3
TGL (mmol/L)	0.3 ± 0.1	0.8 ± 0.1	0.4 ± 0.1	0.3 ± 0.1	0.5 ± 0.1	0.9 ± 0.2	0.5 ± 0.2	0.5 ± 0.1	0.3 ± 0.1	0.7 ± 0.3	0.5 ± 0.2	0.3 ± 0.1	0.4 ± 0.1	0.8 ± 0.2	0.5 ± 0.1	0.5 ± 0.1
GLU (mmol/L)	5.7 ± 1.0	6.3 ± 0.4	5.3 ± 0.4	5.4 ± 0.3	6.1 ± 0.3	6.5 ± 0.4	6.2 ± 0.2	6.2 ± 0.4	5.7 ± 0.3	5.9 ± 0.3	5.8 ± 0.4	5.9 ± 0.4	5.8 ± 0.2	6.0 ± 0.1	5.8 ± 0.3	5.9 ± 0.4
CK (U/L)	343 ± 117	258 ± 35	317 ± 82	317 ± 34	246 ± 32	209 ± 48 *	227 ± 38	220 ± 43	226 ± 31	252 ± 41	260 ± 88	221 ± 39	219 ± 27	186 ± 21	267 ± 48	256 ± 57
K (mmol/L)	5.5 ± 0.3	4.8 ± 0.2	5.4 ± 0.4	5.4 ± 0.2	4.9 ± 0.4	4.6 ± 0.2	5.0 ± 0.3	4.9 ± 0.3	4.9 ± 0.3	4.6 ± 0.3	4.9 ± 0.3	4.8 ± 0.3	4.8 ± 0.2	4.6 ± 0.2	5.0 ± 0.3	4.9 ± 0.2
NA (mmol/L)	153 ± 2	149 ± 1	153 ± 1	152 ± 2	150 ± 1	150 ± 1 *	150 ± 1	150 ± 1	150 ± 1	149 ± 2	150 ± 1	150 ± 2	150 ± 1	146 ± 2	151 ± 1	149 ± 3
CL (mmol/L)	112 ± 2	113 ± 2	112 ± 1	111 ± 1	114 ± 1	113 ± 2	114 ± 1	113 ± 2	114 ± 1	112 ± 2	115 ± 2	114 ± 2	114 ± 1	113 ± 1	116 ± 1	114 ± 3
GGT (U/L)	4.4 ± 2.0	4.1 ± 0.9	4.4 ± 1.9	4.1 ± 1.7	5.8 ± 2.5	3.3 ± 1.8	5.0 ± 1.8	4.9 ± 3.4	6.0 ± 1.7	5.6 ± 1.0 *	5.6 ± 1.8	4.6 ± 2.1	3.8 ± 2.7	5.8 ± 2.6	3.9 ± 2.3	4.9 ± 0.9
CA (mmol/L)	2.6 ± 0.1	2.9 ± 0.1	2.6 ± 0.1	2.5 ± 0.1	2.7 ± 0.1	2.9 ± 0.1	2.7 ± 0.1	2.7 ± 0.1	2.7 ± 0.1	2.8 ± 0.1	2.7 ± 0.1	2.7 ± 0.1	2.8 ± 0.1	2.8 ± 0.1	2.8 ± 0.1	2.7 ± 0.1
PHOS (mmol/L)	2.4 ± 0.2	2.6 ± 0.2	2.3 ± 0.2	2.3 ± 0.3	2.4 ± 0.2	2.4 ± 0.2	2.4 ± 0.2	2.4 ± 0.3	2.3 ± 0.2	2.3 ± 0.2	2.2 ± 0.1 *	2.3 ± 0.2	2.1 ± 0.2	2.2 ± 0.1	2.2 ± 0.1	2.2 ± 0.3

Data were expressed as mean ± SD (*n* = 4). * *p* < 0.05, ** *p* < 0.01, vs. CTL group.

**Table 4:** Pharmacokinetic parameters of Deg-AZM in the plasma of Beagle dogs following repeated oral administration at different doses. Data were expressed as the means ± SD (*n* = 4).

Groups Parameters		5 mg/kg		15 mg/kg		50 mg/kg	
		Day1	Day28	Day1	Day28	Day1	Day28
Male	AUC_0-t_ (μg/mL*h)	5.2 ± 2.1	7.6 ± 6.6	10.3 ± 3.7	17.0 ± 5.7	25.1 ± 4.5	42.0 ± 9.8
	T_1/2_ (h)	1.1 ± 0.15	1.5 ± 0.5	1.2 ± 0.1	1.3 ± 0.1	1.3 ± 0.1	1.4 ± 0.2
	T_max_ (h)	0.70 ± 0.41	0.65 ± 0.34	0.6 ± 0.3	0.6 ± 0.3	0.6 ± 0.3	0.5 ± 0.0
	C_max_ (μg/mL)	2.5 ± 0.82	2.6 ± 1.3	4.1 ± 1.2	6.1 ± 1.7	10.7 ± 2.4	14.7 ± 2.9
Female	AUC_0-t_ (μg/mL*h)	5.4 ± 2.0	8.0 ± 4.7	12.7 ± 5.6	16.3 ± 8.2	45.4 ± 11.6	60.6 ± 15.7
	T_1/2_ (h)	1.1 ± 0.33	4.5 ± 7.0	1.2 ± 0.1	1.3 ± 0.2	1.8 ± 0.2	1.7 ± 0.2
	T_max_ (h)	0.70 ± 0.27	0.60 ± 0.22	0.5 ± 0.1	0.5 ± 0.0	0.9 ± 0.7	0.4 ± 0.1
	C_max_ (μg/mL)	2.6 ± 1.0	3.2 ± 1.9	5.3 ± 1.7	5.7 ± 2.3	12.3 ± 3.0	18.4 ± 3.4

## Data Availability

The original contributions presented in this study are included in the article/Supplementary Material. Further inquiries can be directed to the corresponding author(s).

## Supplementary Materials

The following supporting information can be downloaded at: https://www.mdpi.com/article/10.3390/biomedicines13092180/s1, Figure S1: Pharmacodynamic evaluation of Deg-AZM in relieving atropine induced constipation model.

## Abbreviations

The following abbreviations are used in this manuscript:

5-HT	5-Hydroxytryptamine
A/G	albumin-to-globulin ratio
ALB	albumin
ALP	alkaline phosphatase
ALT	alanine aminotransferase
APTT	activated partial thromboplastin time
AST	aspartate aminotransferase
AUC	area under the concentration-time curve
AZM	azithromycin
BASO	basophil count
BUN	blood urea nitrogen
cGMP	cyclic guanosine monophosphate
CHOL	total cholesterol
CK	creatine kinase
ClC	chloride channel activators
C_max_	peak concentration
CMC-Na	carboxymethylcellulose sodium
CREA	creatinine
Deg-AZM	deglycosylated azithromycin
ECG	electrocardiogram
EOS	eosinophil count
EOS%	eosinophil percentage
F-actin	filamentous actin
FIB	fibrinogen
G-actin	globular actin
GC-C	guanylate cyclase-C
GGT	gamma-glutamyl transferase
GLO	globulin
GLU	glucose
H&E	hematoxylin and eosin
HCT	hematocrit
HED	human equivalent dose
hERG	human ether-à-go-go-related gene
hERG-HEK293	The stably transfected hERG-expressing cell line
HGB	hemoglobin concentration
HR	heart rate
LD_50_	median lethal dose
LYM	lymphocyte count
LYM%	lymphocyte percentage
MCH	mean corpuscular hemoglobin
MCHC	mean corpuscular hemoglobin concentration
MCV	mean corpuscular volume
MLD	minimum lethal dose
MONO	monocyte count
MONO%	monocyte percentage
MRSD	maximum recommended starting dose
NBF	neutral buffered formalin
NEU	neutrophil count
NEU%	neutrophil percentage
NOAEL	no observed adverse effect level
PDW	platelet distribution width
PLT	platelet count
PT	prothrombin time
QTc	QT interval
RBC	red blood cell count
RDW	red cell distribution width
RETIC%	reticulocyte percentage
STC	slow transit constipation
T_1/2_	terminal elimination half-life
TBIL	total bilirubin
TGL	triglycerides
Tmax	time to maximum concentration
TP	total protein
TT	thrombin time
WBC	white blood cell count
