# Supplementary material for: Preclinical Safety Profile of Deg-AZM, a Clinical-Stage New Transgelin Agonist: hERG Inhibition Study In Vitro, Cardiovascular–Respiratory Pharmacology, and Single/Repeated-Dose Toxicity in Beagle Dogs
Source: Biomedicines. 2025 Sep 6;13(9):2180. doi: 10.3390/biomedicines13092180 (PMC12467691; doi:10.3390/biomedicines13092180)
Supplement: Supplementary file 1 [file biomedicines-13-02180-s001.zip › biomedicines-3793397-supplementary.pdf]

1. Pharmacodynamic evaluation of Deg-AZM in relieving atropine-induced constipation model

In the study investigating the therapeutic effect of Deg-AZM on atropine-induced constipation in mice, 72 mice were randomly assigned to six groups (n = 12 per group, housed two per cage): normal control (CTL), model control (Model), positive control (Prucalopride), and Deg-AZM treatment groups at 5, 10, and 15mg/kg. All groups received daily oral administration for two consecutive days. As shown in **Figure S1**, Deg-AZM administration significantly improved fecal dry weight, wet weight, and feces number compared to the Model group. During the 0–4h post-dosing period, all Deg-AZM dose groups exhibited significant increases in fecal wet weight, dry weight, and feces number. From 0–8h post-dosing, significant increases in fecal wet weight and feces number were observed across all dose groups, with the 10 and 15mg/kg Deg-AZM groups additionally showing significant increases in dry weight. The Prucalopride group demonstrated significant increases in all three parameters during both 0–4h and 0–8h intervals. The carbon powder propulsion rate in the Model group was 41.9%, significantly lower than the CTL group. Deg-AZM treatment at 5, 10, and 15mg/kg increased propulsion rates to 48.8%, 55.6%, and 61.3%, respectively, all significantly higher than the Model group. Notably, the 10 and 15mg/kg Deg-AZM groups achieved higher propulsion rates (55.6% and 61.3%) than the Prucalopride group (53.5%). Deg-AZM significantly promoted intestinal motility in atropine-induced

constipated mice, demonstrating marked efficacy with an effective dose of 5 mg/kg.

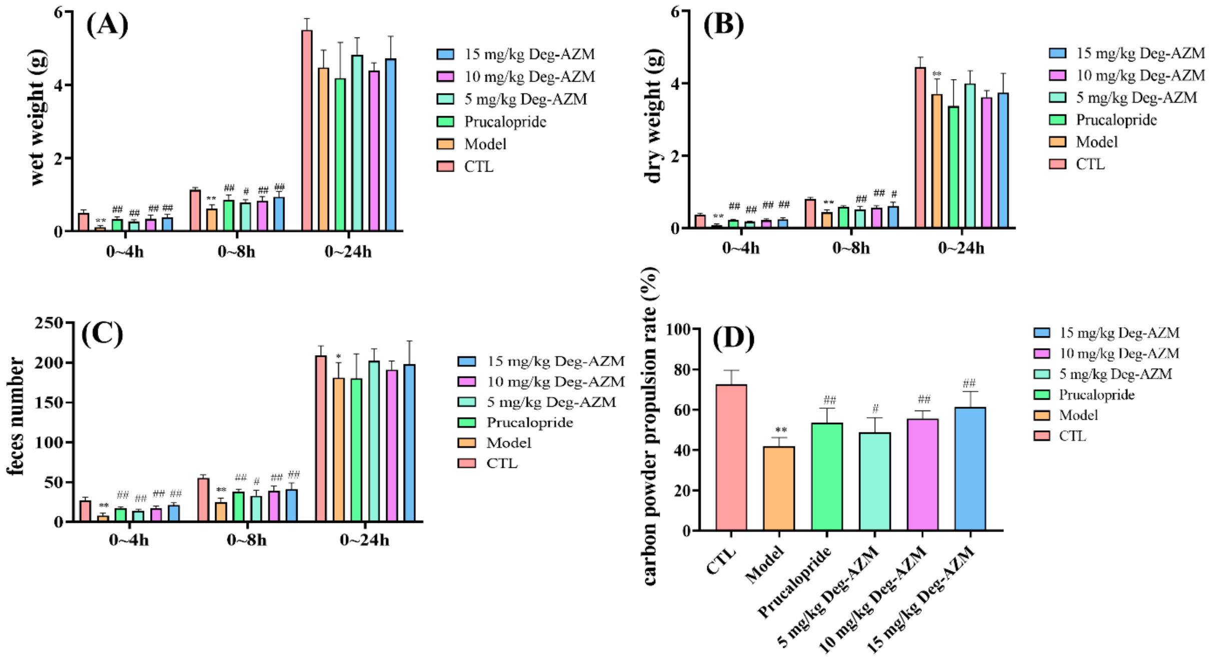

Figure S1. Pharmacodynamic evaluation of Deg-AZM in relieving atropine-induced constipation model. (**A**) The mass of wet feces at different time post administration, $n = 6$; (**B**) the mass of dry feces at different time post administration, $n = 6$; (**C**) feces number at different time post administration, $n = 6$; (**D**) carbon powder propulsion rate, $n = 12$. Data were presented as the mean ± SD, $n = 6$. * $p < 0.05$; ** $p < 0.01$ compared to the CTL group. $^{\#}$ $p < 0.05$; $^{\#\#}$ $p < 0.01$ compared to the Cis group. All p values in F test to compare variances were greater than 0.05.
